# Linkage analysis of adult height with parent-of-origin effects in the Framingham Heart Study

**DOI:** 10.1186/1471-2156-4-S1-S76

**Published:** 2003-12-31

**Authors:** Nandita Mukhopadhyay, Daniel E Weeks

**Affiliations:** 1 Department of Human Genetics, Graduate School of Public Health, University of Pittsburgh, Pittsburgh, Pennsylvania, US; 2 Department of Biostatistics, Graduate School of Public Health, University of Pittsburgh, Pittsburgh, Pennsylvania, USA

## Abstract

Current linkage analysis methods for quantitative traits do not usually incorporate imprinting effects. Here, we carried out genome-wide linkage analysis for loci influencing adult height in the Framingham Heart Study subjects using variance components while allowing for imprinting effects. We used a sex-averaged map for the 22 autosomes, while chromosomes 6, 14, 18, and 19 were also analyzed using sex-specific maps. We compared results from these four analyses: 1) non-imprinted with sex-averaged maps, 2) imprinted with sex-averaged maps, 3) non-imprinted with sex-specific maps, and 4) imprinted with sex-specific maps. We found four regions on three chromosomes (14q32, 18p11-q21, 18q21-22, and 19q13) with LOD scores above 2.0, with a maximum LOD score of 3.12, allowing for imprinting and sex-specific maps, at D18S1364 on 18q21. While we obtained significant evidence of imprinting effects in both the 18p11-q21 and 19q13 regions when using sex-averaged maps, there were no significant differences between the imprinted and non-imprinted LOD scores when we used sex-specific maps. Our results illustrate the importance of allowing for gender-specific effects in linkage analyses, whether these are in the form of gender-specific recombination frequencies, or in the form of imprinting effects.

## Background

Genomic imprinting is the inactivation of a genomic segment depending on the sex of the parent from which it was inherited. Imprinting may play an important role in the inheritance of complex traits in humans. It has been shown by previous studies that incorporation of imprinting into linkage analysis may improve the power of variance component methods for detecting quantitative trait loci [1,2]. However, Shete and Amos [1] advise caution in using imprinted analyses, since they require larger sample sizes than non-imprinted analyses. This difference increases as the distance from the quantitative trait locus increases, and as the imprinting effect decreases [1]. On the other hand, where there is a large imprinting effect, using non-imprinted analysis may reduce power to detect linkage.

We decided to analyze linkage of normal adult height because imprinted genes are involved in growth and development [3]. Further, height is easily and reliably measured, and stable within a certain age range. Height is also highly heritable, with *h*^2 ^values ranging from 0.5 to 0.8 [4-8]. Although our analysis cannot prove that incorporation of imprinting improves linkage analysis, it has the prospect of identifying new regions linked to height. Since sex-specific maps may be more appropriate in this type of analysis [1], we selected the four chromosomes that produced the highest imprinting LOD scores with sex-averaged maps, and reanalyzed them using sex-specific maps.

## Methods

### Subjects

The Framingham Heart Study, which started in 1948, is a longitudinal study designed to evaluate the multifactorial nature of cardiovascular disease. The original cohort, collected in 1948, contained adult members from about two-thirds of the homes in Framingham, Massachusetts. In 1971, a second offspring cohort was included in the study, about three-fifths of which are the biological offspring of the original cohort. A more detailed description of these two cohorts is available in previous publications [9,10]. We analyze here the Framingham Heart Study data as made available to the Genetic Analysis Workshop 13. Our research was approved by the University of Pittsburgh Institutional Review Board (IRB protocol number 020481).

For both cohorts, observers measured and recorded multiple anthropomorphic measurements, at periodic examinations over the duration of the study, at two-year intervals for the original cohort, and at four-year intervals for the offspring cohort. Height measurements were taken with participants standing erect, with heads in the Frankfort plane. For the original cohort, height was measured at biennial examination 1 (in 1948), 5, 10, 13, and then at every subsequent examination onwards. For the offspring cohort, there was approximately an 8-year gap between the first exam (in 1971–1974) and the second exam; subsequent exams were given every 4 years.

### Data cleaning and phenotype definition

We examined the 2885 individuals who had multiple height measurements. Each individual's height measurements were compared in a pair-wise fashion for deviations greater than 2.0 inches. Where a single unique measurement was deviant from all others, this measurement was removed, along with its corresponding examination age. There were 22 such individuals. If we were unable to identify a unique deviant, we set that individual's height and age to unknown for the rest of the analysis. Only 12 individuals were zeroed out in this fashion.

For each individual with known height measurements, we first computed a mean adult height measurement between the ages of 20–60 years, and a mean adult age of examination. Linear regression was performed to eliminate gender and age effects. Since there is evidence of change in the population distribution of height in the last few decades, we also regressed mean height against decade-of-birth for each individual. The residuals resulting from the linear regression of height against gender, mean-age, and decade-of-birth were converted into z-scores, which were used as the trait phenotype for linkage analysis.

### Variance components with parent-of-origin effects

In order to incorporate imprinting, we have to include separate major-gene components for each parent. If we assume purely additive variance, the variance-covariance matrix Ω is:

Ω = Π_MO_σ^2^_aMO _+ Π_FA_σ^2^_aFA _+ 2Φσ^2^_G _+ Iσ^2^_e_,

where Π_MO _is a matrix containing the estimated proportion of maternal alleles shared identically by descent (IBD), Π_FA _is a matrix containing the estimated proportion of paternal alleles shared IBD, Φ is the kinship matrix, and I is an identity matrix. Here, we are partitioning the variance into four components: an additive component (σ^2^_aMO_) due to the effect of a maternally derived allele linked to the region of interest, an additive component (σ^2^_aFA_) due to the effect of a paternally derived allele, a polygenic component (σ^2^_G_), and an environmental effect (σ^2^_e_). As a simplifying assumption, we ignore the effects of shared environment. We computed five different likelihoods on our pedigree data:

L(σ^2^_aMO _= 0, σ^2^_aFA _= 0, σ^2^_G_, σ^2^_e_): No major gene effect.     (1)

L(σ^2^_aMO _= σ^2^_aFA_, σ^2^_G_, σ^2^_e_): Non-imprinted, where σ^2^_aMO _and σ^2^_aFA _are constrained to be equal.     (2)

L(σ^2^_aMO _= 0, σ^2^_aFA_, σ^2^_G_, σ^2^_e_): Complete maternal imprinting, σ^2^_aMO _= 0     (3)

L(σ^2^_aMO_, σ^2^_aFA _= 0, σ^2^_G_, σ^2^_e_): Complete paternal imprinting, σ^2^_aFA _= 0     (4)

L(σ^2^_aMO_, σ^2^_aFA_, σ^2^_G_, σ^2^_e_): Imprinted, i.e. σ^2^_aMO_, σ^2^_aFA_≥ 0     (5)

We tested for linkage at each marker for equations (2–5) by comparing its log-likelihood against that of the polygenic equation (1) using the likelihood ratio test. LOD scores were also computed by dividing the likelihood ratio by 2ln(10). We also examined evidence for imprinting by comparing the log-likelihood of the non-imprinted model to that of the imprinted model.

### Linkage analysis

Linkage analysis was performed on 330 extended pedigrees containing 4692 individuals. SIMWALK2 version 2.83 [11] was used for multipoint IBD estimation [12] on the complete extended pedigree structures. SIMWALK2 returned sharing estimates for the 15 possible detailed identity states, from which we derived estimates of maternal and paternal IBD sharing for all the parent-offspring pairs and sibling pairs. The variance-component imprinting models described above are currently only applicable to nuclear families; therefore, the extended pedigrees were broken down into their nuclear components for the variance components analyses. Variance components linkage analysis was performed using FISHER [13] driven by our own imprinting module, which computes the likelihoods described above. All map distances are in Kosambi centimorgans.

## Results

### Regression and phenotype distribution

Height is dependent on gender, decade-of-birth, and age as follows: β_gender _= -5.40, β_decade-of-birth _= 0.045, and β_age _= -0.01, with R^2 ^= 0.5521. Gender and decade-of-birth effects are significant at the 0.01 level. The residuals are distributed normally with a mean of 0.00, standard deviation of 2.58 inches, skewness 0.094, and kurtosis = -0.174. The Shapiro-Wilkes test statistic is 0.99 with a *p*-value of 0.08. After conversion to z-scores, there are only five outliers, two individuals with z-scores less than 3.0, and three with scores larger than 3.0. These individuals were excluded from the linkage analysis.

### Linkage analysis results

**Figure 1:**
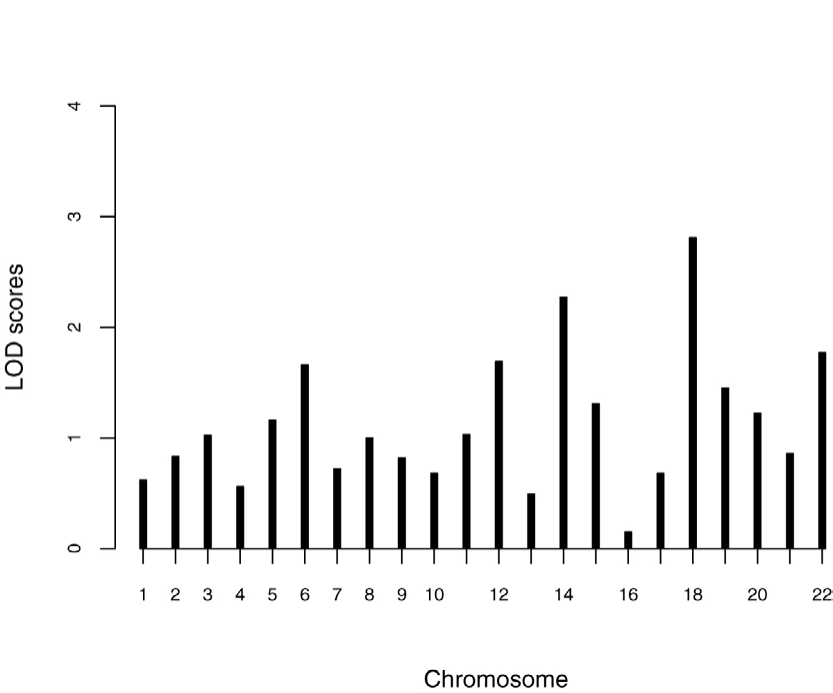
**Highest imprinting LOD score per chromosome **Chromosomes analyzed with sex-averaged maps.

**Figure 2:**
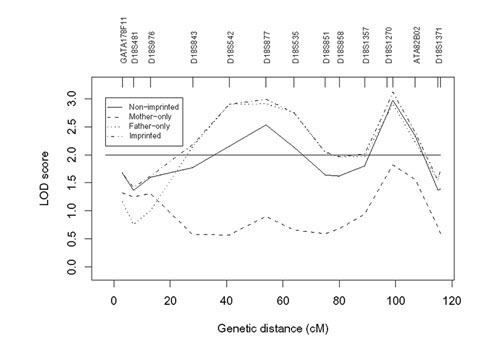
**Chromosome 18 LOD scores with sex-specific maps **LOD scores at each marker have been joined to produce continuous curves. Map distances on X-axis are from the sex-averaged map.

**Figure 3:**
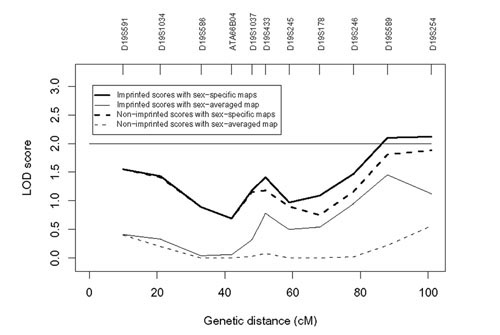
**Chromosome 19 LOD scores for sex-specific and sex-averaged maps **Map distances on X-axis from the sex-averaged map.

We considered markers with LOD scores at or above 2.0 as being of possible interest (Figure 1). (However, note that for non-imprinted LOD scores, a LOD of 2 has an asymptotic *p*-value of approximately 0.0012, while for imprinted LOD scores, a LOD of 2 has an asymptotic *p*-value of approximately 0.0037.) Conventional analyses (e.g., not modelling sex-differences or imprinting) identify two regions, 14q32.2 and 18q21.3-22.1 (Table 1). Allowing for imprinting while using sex-averaged maps identifies an additional region, 18p11-q21.1, with a LOD score above 2.0 (Table 1). Using sex-averaged maps, we observe significant evidence of imprinting in two regions, 18p11-18q21.1 and 19q13.3-19q13.4. Three regions continue to give linkage signals when we allow for sex-specific maps without imprinting, although the 14q32.2 maximum LOD score drops to 1.99 (Table 1). When we allow for imprinting and sex-specific maps, we add a fourth region, 19q13.3-13.4, with LOD scores above 2.0, and we obtain our highest LOD score of 3.12 at D18S1364 (Table 1, Figure 2). When we use sex-specific maps, we no longer see any significant evidence of imprinting when we contrast the imprinting LOD score with the non-imprinting LOD score (Table 1). However, at D18S542 we have significant evidence of maternal imprinting due to the large difference between the imprinting LOD score and the mother-only imprinting LOD score (Figure 2). Region 19q13.3-19q13.4 has significant imprinting effects at D19S589 if sex-averaged maps are used (Table 1, Figure 3). However, these imprinting effects are not significant when sex-specific maps are used-note how close to each other the non-imprinted and imprinted LOD scores are at D19S589 when sex-specific maps are used (Figure 3).

## Discussion

Several genome-wide linkage analysis studies have attempted to pinpoint the genetic factors that control height. A study on Pima Indians showed evidence of a locus on chromosome 20 [14]. Other studies found evidence of linkage of height on chromosomes 6, 7, 12, and 13 [15], and on chromosomes 7 and 9 [16]. More recently, evidence for linkage was found on chromosome 3 [17]; and evidence for linkage was observed on chromosomes 6q25, 9p1, and 12q1 [18]; and on chromosomes 5q31, Xp22, and Xq25 [19]. None of these studies considered imprinting effects or used sex-specific genetic maps. Our study was unable to replicate any of these regions, however, we obtained a LOD score of 1.94 at marker D6S503 in the 6q27 region with imprinting on sex-specific maps, close to regions linked to height in two other studies [15,18]. It is interesting to note that the estrogen receptor 1 (*ESR1*) gene is close to this region.

We explore here the impact of carrying out linkage analyses while allowing for sex-specific map differences and imprinting. If the male recombination fraction (θ_m_) is not equal to the female recombination fraction (θ_f_), then a test that assumes θ_m _= θ_f _will be invalid. Furthermore, in a region of true linkage, such map misspecification results in loss of power to detect linkage [20]. Hanson et al. [2] found by simulation (as confirmed by analytical analyses by Shete and Amos [1]) that testing for imprinting effects while erroneously assuming θ_m _= θ_f _can increase the type I error rate of the imprinting LOD score, but that these increases are modest if the female:male map-distance ratio is ≤ 5:1. In our study, using sex-averaged maps, we obtained significant evidence of imprinting in precisely those regions with large differences between the male and female genetic distances (Table 1). After we accounted for the sex-specific map differences, then we no longer had significant evidence for imprinting effects, and the most parsimonious explanation of our data is linkage without any imprinting. The linkage signals are strongest when we properly model the male and female recombination fractions by using sex-specific maps. However, it is important to point out that detection of imprinting effects requires large sample sizes, and that region 18p11-q21, where our highest LOD scores were obtained, is known to contain imprinted genes [21].

It may not be appropriate to ignore dominance effects as we have in our variance components models. In addition, Spencer [22] shows that the covariance should include an interaction between the dominance and additive terms in the presence of imprinting.

## Conclusions

Most previous variance components-based analyses [23,24] testing for linkage while allowing for imprinting have been limited in that sex-specific maps have not been used in the analyses, mainly due to computational difficulties. The combined use of SIMWALK2 [11] and FISHER [13] permits incorporation of sex-specific maps into the analyses. As Shete and Amos [1] point out, "in the imprinting-testing model, it is important to include this difference between the male and female recombination fractions."

## Figures and Tables

**Table 1:** Regions with LOD scores ≥ 2.0^A^

		**Genetic distances (cM)**			**Sex-Averaged Map LOD Scores**		**Sex-Specific Map LOD Scores**	
		
**Chromosome**	**Marker, Female:Male Ratio**	**Sex-Average**	**Female**	**Male**	**Non-Imprinted**	**Imprinted**	**Non-Imprinted**	**Imprinted**
14q32.2								
	D14S1434				0.24	0.32	0.17	0.23
	0.53	13	9	17				
	D14S1426				2.01	2.27	1.99	2.01
18p11-18q21.1								
	D18S976				0.26	0.31	1.60	1.62
	0.67	15	12	18				
	D18S843				0.23	0.97	1.77	2.18
	2.38	13	19	8				
	D18S542				0.57	1.95^B^	2.15	2.90
	25.00	13	25	1				
	D18S877				1.26	2.08	2.53	2.99
	9.00	10	18	2				
	D18S535				1.00	1.86^B^	2.13	2.75
	2.8	11	14	5				
	D18S851				0.22	1.10^B^	1.64	2.05
	1.80	5	9	5				
	D18S858				0.23	0.93	1.62	1.97
18q21.3-18q22.1								
	D18S858				0.23	0.93	1.62	1.97
	7.50	9	15	2				
	D18S1357				0.50	1.09	1.80	2.01
	1.50	8	9	6				
	D18S1270				1.99	2.61	2.72	2.87
	1.00	2	3	3				
	D18S1364				2.26	2.81	2.97	3.12
	2.00	8	10	5				
	ATA82B02				1.26	1.51	2.33	2.40
	1.71	9	12	7				
	D18S1371				0.28	0.33	1.37	1.55
19q13.3-19q13.4								
	D19S246				0.02	0.95	1.16	1.47
	0.46	10	6	13				
	D19S589				0.22	1.45^B^	1.81	2.10
	0.29	13	6	21				
	D19S254				0.56	1.12	1.88	2.12

^A^Markers for which any LOD score was greater than 2.0. ^B^Imprinted locus (significance level ≤ 0.05).
